# Recurrent Cryptogenic Stroke in a Patient With Left Atrial Septal Pouch

**DOI:** 10.7759/cureus.24241

**Published:** 2022-04-18

**Authors:** Arshan Khan, Maria Riasat, Moiz Ehtesham, Dominic Brink, Kelash Rai, Muhammad Haseeb, Warisha Khan, Rami Zein

**Affiliations:** 1 Internal Medicine, Ascension St. John Hospital, Detroit, USA; 2 Internal Medicine, Icahn School of Medicine at Mount Sinai, New York, USA; 3 Internal Medicine, Albany Medical Center, Albany, USA; 4 Cardiology, Ascension St. John Hospital, Detroit, USA; 5 Internal Medicine, Wayne State University School of Medicine, Rochester Hills, USA; 6 Internal Medicine, Bahria International Hospital, Lahore, PAK; 7 Internal Medicine, Jinnah Hospital, Lahore, PAK; 8 Internal Medicine, Faisalabad Medical University, Faisalabad, PAK

**Keywords:** treatment of left atrial septal pouch, pfo, double atrial septum, left atrial septal pouch, cryptogenic stroke

## Abstract

The incomplete fusion of the septum primum and septum secundum results in the formation of the left atrial septal pouch (LASP). The clinical significance of this entity is a matter of controversy; however, it may act as a nidus for thrombus formation. We report a case of a 57-year-old male who was brought to the hospital by his girlfriend due to his bizarre behavior and confusion for one day. The initial workup for his altered mental status did not yield a diagnosis. The patient was admitted for further workup, which included an MRI of the brain that showed numerous very small-sized foci of restricted diffusion involving bilateral cerebral and cerebellar hemispheres consistent with thromboembolic infarct. The patient did not receive a tissue plasminogen activator (TPA) as he was out of the window for TPA. Transthoracic echocardiogram (TTE) with bubble study did not show patent foramen ovale (PFO) or atrial septal defect (ASD). ECG and telemetry showed normal sinus rhythm and no atrial fibrillation. A transesophageal echocardiogram (TEE) was obtained to find the source of the thromboembolic stroke. TEE discovered a 22 x 8-mm cystic structure in the interatrial septum consistent with a LASP. We hypothesize that the LASP may be a risk factor for cryptogenic stroke. Further research is needed to determine the prevalence of atrial septal pouch (ASP) in the general population, its clinical significance, and guidelines for treatment implications.

## Introduction

The left atrial septal pouch (LASP) is a common anatomical variant formed by the incomplete fusion of the septum primum and septum secundum at the interatrial septum [1]. The LASP cavity may increase the risk of blood clot formation and can be a potential source of systemic thromboembolism and ischemic stroke [2-4].

The LASP is commonly diagnosed on a transesophageal echocardiogram (TEE) with a bubble study [5]. Despite some evidence of the LASP involvement in the pathogenesis of cardioembolic stroke, the question of the clinical significance of the LASP remains unsolved [6]. In this report, we present the case of a patient with recurrent thromboembolic cryptogenic stroke who was found to have a LASP on TEE.

## Case presentation

A 57-year-old male was brought to the hospital by his girlfriend on account of his bizarre behavior and confusion for one day. He was also noted to have stool incontinence, but there was no report of any seizure or seizure-like activity. His medical history was significant for ischemic cerebrovascular accident (CVA) without residual deficit, heart failure with a reduced ejection fraction (EF) of 30% with a LifeVest, moderate pulmonary hypertension, coronary artery disease, non-insulin-dependent diabetes mellitus, essential hypertension, chronic alcoholism, and vitamin B12 deficiency.

In the emergency room, the patient's blood pressure was 155/78 mmHg, and the rest of the vitals were within normal limits. The patient appeared to be confused; there was mild ataxia with gait; otherwise, the rest of the physical examination was unremarkable, including a complete neurological examination. Laboratory workup revealed thrombocytopenia with a platelet count of 136 K/mcL, blood urea nitrogen (BUN) of 81 mg/dl, creatinine of 1.37 mg/dl, anion gap of 16 mEq/L, alanine aminotransferase (ALT) of 53 IU/L, aspartate aminotransferase (AST) of 49 IU/L, and pro-B-type natriuretic peptide (proBNP) of 4570 pg/ml. Urinalysis was unremarkable; serum ammonia and the lactic acid level were within normal limits. The urine drug screen was negative. Serum ethanol, acetaminophen, and salicylate levels were not elevated. A chest X-ray was obtained, which was unremarkable. CT head without contrast was obtained, which did not reveal any acute intracranial findings. An ECG showed sinus rhythm, left axis deviation, left anterior fascicular block, and QTc prolongation of 502 ms.

**Figure 1:**
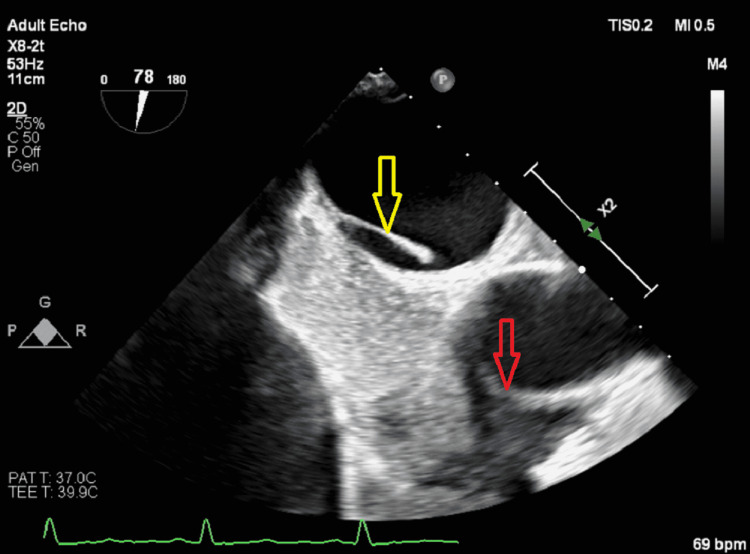
TEE bicaval view with saline agitated study showing a LASP (yellow arrow) and saline bubbles in the right atrium (red arrow) but not crossing into the right atrium TEE: transesophageal echocardiogram; LASP: left atrial septal pouch

**Figure 2:**
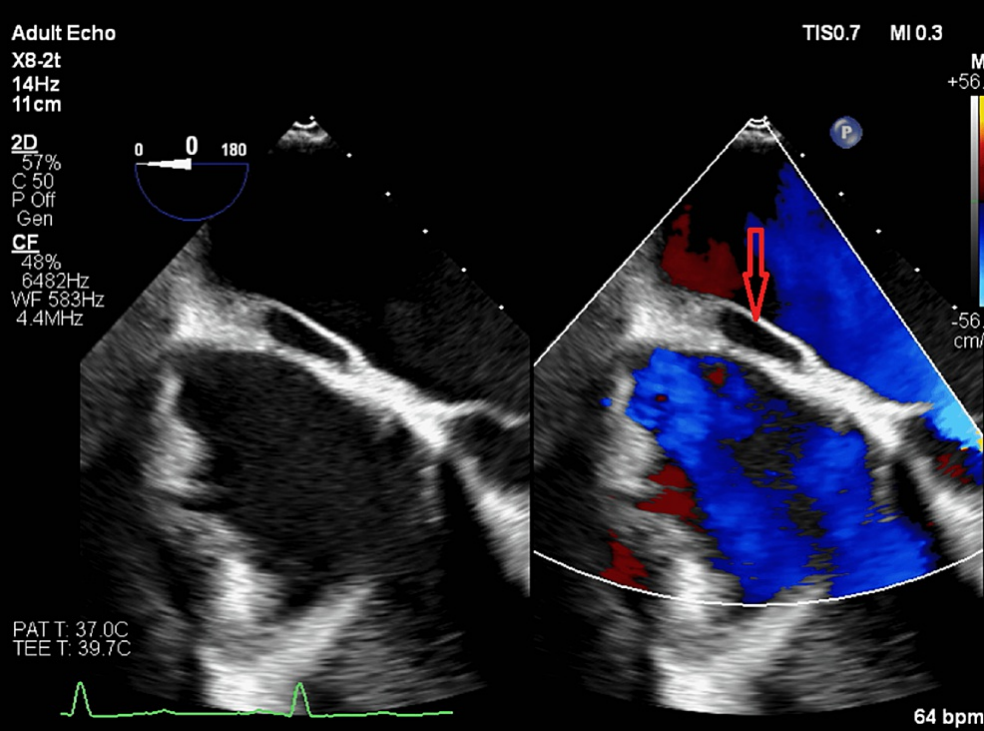
TEE four-chamber view at 0 degrees with a focus on the atrial septum (red arrow). The color Doppler flow shows no communication with the pouch TEE: transesophageal echocardiogram

The patient was admitted for the workup of altered mental status with an MRI of the brain with and without contrast and neurology consultation. The next day, the MRI of the brain showed multiple very small-sized foci of restricted diffusion involving bilateral cerebral and cerebellar hemispheres concerning for thromboembolic infarct. The patient did not receive a tissue plasminogen activator (TPA) as he was out of the window for TPA. EEG was also obtained, which did not show any focal or epileptiform foci. TEE with bubble study was obtained as a part of a stroke pathway, which showed EF of 30-35%, severe hypokinesis of the basal to mid inferior, and inferoseptal walls. There was no evidence of thrombus or patent foramen ovale (PFO). There was no atrial fibrillation recorded on telemetry or ECG. Interrogation of the LifeVest also did not demonstrate any atrial fibrillation. At that time, a decision was made to proceed with a TEE and CT aortogram to identify the source of thromboembolism. The CT aortogram was unremarkable. On TEE, a 22 x 8-mm cystic structure was seen in the interatrial septum (Figures 1, 2). After the administration of the contrast agent, there was uptake of the contrast seen within the cystic structure. Most likely, this represented a LASP. In our patient, the LASP may have been a nidus for thrombus formation and recurrent embolic stroke, as no other source of recurrent thromboembolic stroke was found. The patient was discharged to rehab on apixaban, clopidogrel, and atorvastatin for the further prevention of thromboembolic stroke.

## Discussion

During embryological development, the human heart undergoes multiple processes, which include folding, overlapping, and merging. The interatrial septum is one of these sites where septum primum and septum secundum come together and fuse. Due to the complexity of the process, sometimes the two septa do not overlap completely in the entire zone, giving rise to a defect known as the atrial septal pouch (ASP). When ASP opens toward the left atria, it is called a LASP.

ASP is a partial fusion defect, which does not include an interatrial shunt. Total failure of the adhesion would lead to PFO [1,2,7]. The ASP can be a site for blood stasis and thrombus formation with potential embolic complications. It can also trigger atrial fibrillation, perhaps due to the presence of scar tissue in the septal pouch apex. The diagnosis can be made with the aid of imaging techniques such as TTE, TEE, cardiac CT, and cardiac MRI. ASP is poorly visualized in TTE, mainly because of the posterior position of the pouch in comparison to the other structures of the heart. The TEE with agitated saline is the gold standard for the diagnosis of the LASP [8-10].

**Figure 3:**
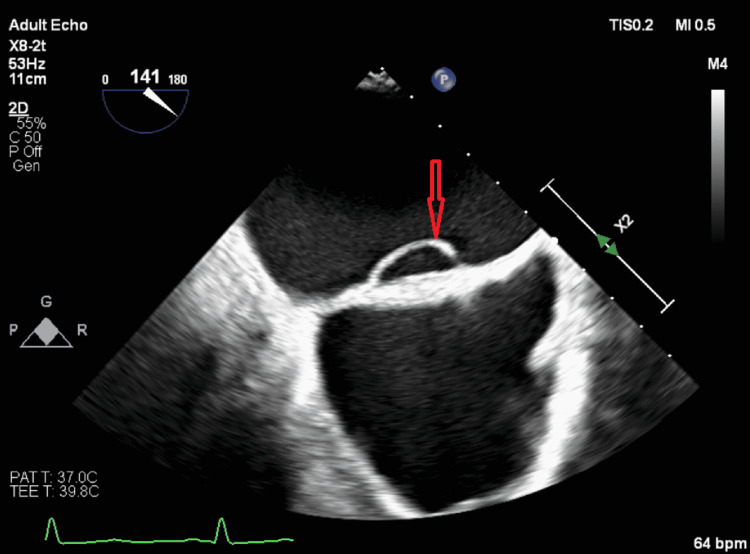
TEE mid-esophageal view at 141 degrees showing a LASP (red arrow) TEE: transesophageal echocardiogram; LASP: left atrial septal pouch

Our patient had a recurrent cryptogenic stroke with evidence of thromboembolic phenomena on MRI. The patient's ECG, telemetry, and LifeVest evaluation were negative for atrial fibrillation. Initial TTE with bubble study did not reveal any defect in the interatrial septum. Eventually, a LASP was diagnosed by TEE (Figure 3). The patient was discharged to rehab on apixaban, clopidogrel, and atorvastatin for the further prevention of thromboembolic stroke.

Approximately 87% of strokes have an ischemic origin (20% cardioembolic events), and 10-30% are considered cryptogenic. It is important to explore other potentially embolismic structures of the left atrium [11-12]. The LASP is one of the potential sites for blood clots formation and can be a potential source of systemic thromboembolism. The other differential diagnosis of the LASP is double atrial septum with a persistent interatrial space [13]. The double atrial septum is an extremely rare congenital anomaly, which has a double-walled atrial septum that distinguishes the midline interatrial chamber between the two atria. This space can communicate with the left atrium via the PFO and with the right atrium through an accessory fenestration [14]. The presence of stasis within the interatrial chamber, created by the restriction of flow between this chamber and the left atrium, may result in thrombus formation and cardioembolic stroke [15].

Currently, there are no specific guidelines for the management of ASP. Some studies have mentioned anticoagulation, while mitral atriotomy was also performed in one case report by removing the septal membrane, which was a nidus for thrombus formation [16]. More research is needed to determine the prevalence of ASP in the general population, its clinical significance, and guidelines for treatment implications [17].

## Conclusions

The LASP is a common anatomical variant formed by incomplete fusion of the septum primum and septum secundum at the interatrial septum. TEE is the diagnostic test of choice for diagnosing the LASP. The LASP cavity may increase the risk of blood clots formation and can be a potential source of systemic thromboembolism and cryptogenic stroke. The LASP should be in the differential diagnosis in patients with cryptogenic stroke. Only a few case reports are available in the literature regarding cryptogenic stroke and the LASP. Our case was unique as our patient had normal sinus rhythm and recurrent thromboembolic cryptogenic stroke. No source of thromboembolism was identified other than the LASP. Currently, there are no specific guidelines for the management of the LASP. Most patients are treated with antiplatelet and anticoagulation without recurrence of stroke. More studies are required to validate a causal relationship between the LASP and cryptogenic stroke and for providing guidelines for treatment implications.
